# Trends in colorectal cancer mortality in hispanics: a SEER analysis

**DOI:** 10.18632/oncotarget.21938

**Published:** 2017-10-19

**Authors:** Afsaneh Barzi, Dongyun Yang, Sayedamin Mostofizadeh, Heinz-Josef Lenz

**Affiliations:** ^1^ Norris Comprehensive Cancer Center, University of Southern California, Los Angeles, CA 90033, USA

**Keywords:** hispanics, SEER, outcome, colorectal cancer, disparity

## Abstract

**Background:**

Colorectal cancer (CRC) mortality among Hispanics is lower than Non-Hispanic Whites (NHW). If Hispanics receive equitable care and achieve the same degree of health benefit, their trend of better survival should be maintained. This study assesses mortality trends among Hispanics overtime to compare their survival improvement with NHW.

**Methods:**

We used the Incidence-Based Mortality database of the Surveillance, Epidemiology, and End Results Program (SEER) to assess the mortality gap, which is defined as the difference in stage-specific mortality between NHWs and Hispanics, and currently has an advantage for Hispanics.

**Results:**

There is a statistically significant disparity in stage-specific mortality of CRC by race, with a higher proportion of deaths from metastatic disease among Hispanics than NHW (48% vs. 44% respectively). Comparing the time intervals of 2000-2005 and 2006-2011, mortality was reduced by 15.1% for NHWs and 5.9% for Hispanics, resulting in a narrowing of the mortality gap. The mortality gap between Hispanics and NHW is tapering overtime for every stage of the disease, reflecting that Hispanics have a disparity in CRC mortality.

**Conclusion:**

The mortality gap between Hispanics and NHWs is narrowing, supporting a significantly lower rate of mortality improvement in Hispanics. If the current trend continues, mortality rates in Hispanics will outpace that of NHWs.

## INTRODUCTION

Colorectal cancer is the second most common type of cancer and the third cause of mortality from cancer in the Hispanic population [1]. Compared to non-Hispanic Whites (NHW), Hispanics have a lower incidence and mortality from colorectal cancer [2, 3].

The advent of effective screening and improved treatments has reduced mortality related to regional and distant disease, thereby leading to an overall mortality decline from colorectal cancer in the US [2]. However, the rate of decline among Hispanics is less than the rest of the population [2, 4, 5].

According to the United States Census Bureau, Hispanics comprise 17.6% of the US population, making them the largest minority. Moreover, they are the largest growing minority in the US. Projections of the 2014 census suggest that Hispanics are expected to grow to 28.5% of the US population by 2060 [6, 7]. The Hispanic population is also younger than the average US population. Therefore, as the population ages, it is expected that the relative incidence of colorectal cancer and its mortality will rise in the near future.

Given that Hispanics make up a significant proportion of the US population and are a growing minority, it is imperative to explore the patterns of colorectal cancer mortality in this population. Exploring the causes for observed disparities in the mortality rate will help us propose strategies to change the trend in near future.

Compared with the Black population, there is a paucity of publications about colorectal cancer mortality among Hispanics. Hence, reasons for this disparity in outcome are not well explored. Hispanics have lower rates of colorectal cancer screening and higher rates of late stage diagnosis [8, 9, 10]. Access to care plays a significant role in cancer outcomes and is a major factor in health disparities. Hispanics are presumed to have decreased treatment benefits, especially from chemotherapy and targeted agents, and have an uneven experience of toxicities from systemic therapies. This can undermine adequate exposure to newer antineoplastic agents, thereby contributing to disparities in CRC mortality [11]. Furthermore, the enrolment rate of Hispanics in clinical trials is poor, which makes it difficult to directly assess their adherence and benefits from emerging treatment strategies [12].

To better understand the mortality trends among Hispanics, we performed an analysis of stage-specific mortality rates among Hispanics and non-Hispanic Whites (NHW) using the Incidence-Based Mortality Database of the Surveillance, Epidemiology, and End Results program (SEER). Here we present the results of this analysis.

## RESULTS

**Figure 1:**
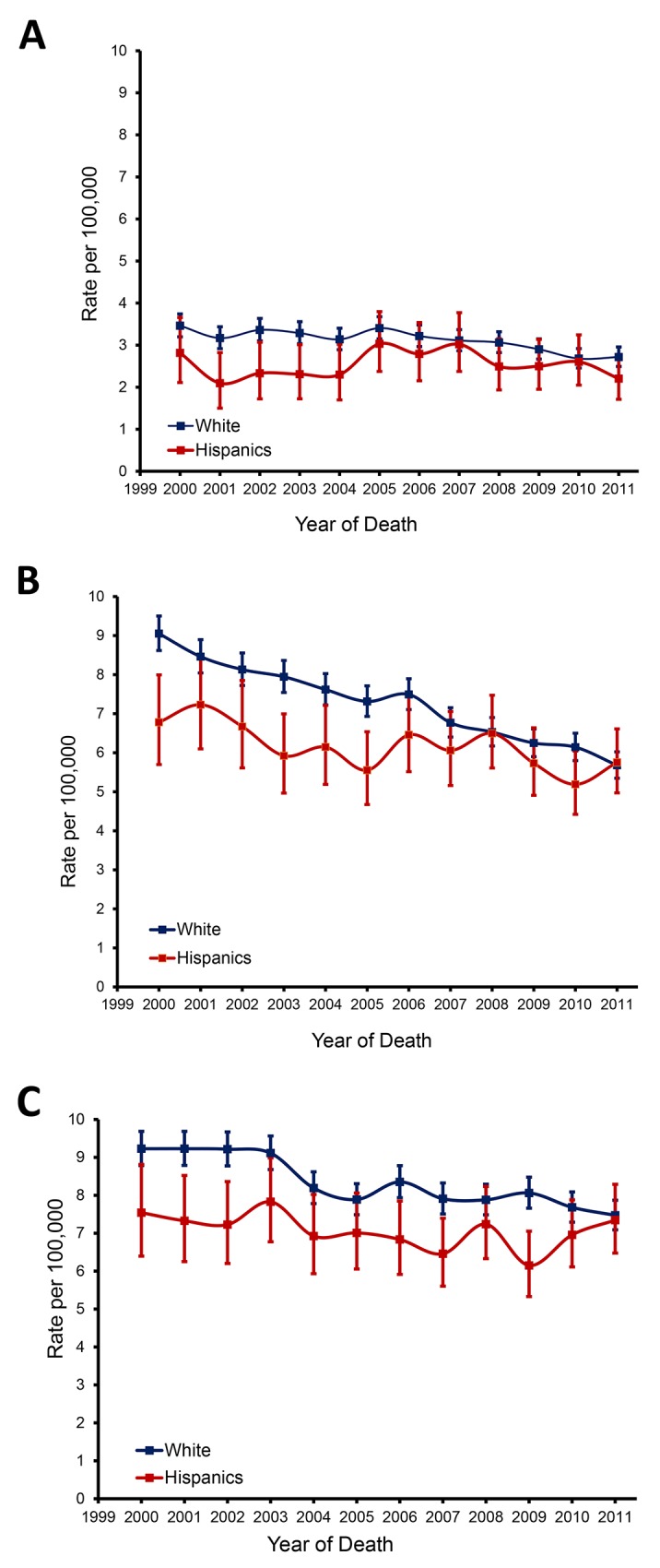
Trends in stage-specific incidence-based mortality in Hispanics and non-Hispanic whites **(A)** localized disease; **(B)** Regional disease; **(C)** distant disease.

We identified 48, 316 total deaths due to colorectal cancer, where 5, 547 occurred in Hispanics and 42,769 occurred in NHWs. Hispanics have lower rates of mortality from colorectal cancer and a mortality advantage. However, the decline in mortality rate is much less in Hispanics than NHWs, and therefore, the advantageous difference in mortality as compared to NHWs is disappearing for Hispanics in all stages of the tumor (Figure 1).

To assess the disparity in the mortality between NHWs and Hispanics, we calculated the mortality gap referred to as “gap” (Table 1). The gap has an advantage for Hispanics, but it is narrowing. This suggests a disproportionate improvement in mortality between the two groups, which is in favor of NHWs. While the mortality of NHWs decreased by 15% between 2000 and 2011, Hispanics experienced only a 6% drop in mortality.

**Table 1:** Changes in stage-specific incidence-based mortality by race and period

	Incidence-based mortality (deaths per 100,000) Year of death in 2000 to 2005				Incidence-based mortality (deaths per 100,000) Year of death in 2006 to 2011				Relative % Change in IBM between 2 periods	
Stage	NHW	Hispanics	Gap (NHW-Hispanics)	Contribution to overall mortality	NHW	Hispanics	Gap (NHW-Hispanics)	Contribution to overall mortality	NHW	Hispanics
Localized	3.3	2.5	0.8	20.6	2.9	2.6	0.4	21.0	-10.9	3.3
Regional	8.1	6.3	1.7	44.1	6.5	5.9	0.5	30.3	-20.1	-6.6
Distant	8.8	7.3	1.5	38.6	7.9	6.8	1.0	59.4	-10.5	-6.1
Unknown	1.1	1.2	-0.1	-3.4	0.8	1.0	-0.2	-10.6	-27.9	-20.4
Overall	21.2	17.3	3.9		18.0	16.3	1.7		-15.1	-5.9

We then calculated the degree of contribution of the gap by stage to the overall mortality (gap across different stages divided by gap for each stage). The disparity between Hispanics and NHWs is greatest for those with regional and distant disease, with distant disease being the major contributor to the overall disparity gap.

To characterize mortality trends over time, we calculated Annual Percent Change (APC). Table 2 reports on the APC in different stage groups between 2000 - 2011. Hispanics with localized and distant disease had no improvement in mortality, while those with regional disease had a lesser degree of improvement in outcome compared to NHW. When analyzed by gender, a significant decline in the mortality of regional disease was observed only in Hispanic males (Table 2).

**Table 2:** Trends in annual percentage change in mortality between 2011 and 2000 to assess the changes in stage specific mortality for CRC in Hispanics and NHWs

Stage	Race	All	Male	Female
Localized	NHW	-1.92^*†^	-0.88/-4.32^**^	-2.15^*^
	Hispanics	0.00^†^	-0.26	0.29
Regional	NHW	-3.81^*†^	-3.94^*^	-3.84^*^
	Hispanics	-1.78^*†^	-1.93^*^	-1.91
Distant	NHW	-1.98^*^	-2.61^*^	-1.40^*^
	Hispanics	-0.75	-0.92	-0.68

(^*^) Significant

(^**^) Stable between 2000-2007 and then significant decline between 2007-2011.

^†^ APC was significant by race.

Likewise, to account for changes in treatment of colorectal cancer between 2000 and 2011, we analyzed the mortality of cohorts in two time periods: 2000-2005 and 2006 -2011 in different SEER stages. The time cut-off is selected based on the visual observation of the graphs in Figure 1 and FDA approval dates for new colorectal cancer agents. As seen in Table 1, the mortality gap is smaller for the later cohort. If this trend continues, the mortality of colorectal cancer in Hispanics will outpace that of NHWs.

We explored the stage at diagnosis and location of the tumor in this population (Table 3) and found a disparity in mortality by stage and location. More Hispanics die from distant disease, whereas more NHWs die from localized disease (p-value<0.001, χ^2^ test). Furthermore, there is a difference in mortality by location between the two populations, with more NHWs dying of right sided disease (p-value<0.001, χ^2^ test). When mortality is assessed by gender, the ratio of males with colorectal cancer dying of the disease was 55% and 51% in Hispanics and NHWs respectively (p-value <0.001, χ^2^ test).

**Table 3:** Characteristics of cases in SEER

	Hispanics (n=5547)	NHW (42769)
Local Stage (%)	792 (14%)	7324 (17%)
Regional Stage (%)	2078 (38%)	16645 (39%)
Distant Stage (%)	2677 (48%)	18800 (44%)
Right-sided tumors (%)	2007 (37%)	18140 (44%)
Left-sided tumors (%)	2026 (38%)	14561 (35%)
Rectal (%)	1290 (24%)	8649 (21%)

## DISCUSSION

Our study unveils a significant disparity in colorectal cancer mortality between Hispanics and NHWs in the US. Notably, our study reveals that the mortality gap for colorectal cancer is closing in favor of NHWs (Table 1). We show that between 2000-2005 and 2006-2011, the mortality from colorectal cancer in NHWs had declined by about 15.1% yet Hispanics only saw a 5.9% mortality decline. This observation highlights a significant disparity in the mortality from colorectal cancer by ethnicity. Moreover, the mortality gap between the two groups is getting smaller over time (from 3.9 in the period of 2000-2005 to 1.7 in the period of 2006-2011) and thus, the disparity in mortality for Hispanics is widening.

To explain the observed trends of mortality by stage, an understanding of treatment options for different stages of the disease is helpful. Management of localized, regional, and distant disease relies upon appropriate use of a combination of several different modalities. For localized colon cancer, cure is achieved through surgical resection. For regional colon cancer and locoregional rectal cancer, treatment entails surgical resection combined with adjuvant chemotherapy and neo-adjuvant radiation therapy. For distant disease, treatment options are limited to systemic chemotherapy, with a small proportion of patients being candidates for extensive surgeries with the goal of cure. Over the past three decades, advancement in surgical techniques for colon cancer resection have resulted in overall better outcomes. Data supports that the same surgery in the hands of a colorectal surgeon results in an improved rate of cure, most likely due to better surgical techniques [13]. Hispanics have lower rates and quality of insurance, therefore, it is plausible that they have limited access to specialized centers (surrogate for high penetration of specialists) resulting in a stagnant rate of mortality [14]. Furthermore, cure for rectal cancer depends on administration of neo-adjuvant radiation therapy in addition to surgery and adjuvant therapy. Therefore, access to care may explain inferior outcomes of rectal cancer in the Hispanic population. Our group had previously published that Hispanics have more delays in administration of adjuvant chemotherapy, which may result in decreased benefit from adjuvant chemotherapy in patients with regional disease [15].

Over the past two decades, use of combination chemotherapy, surgical resection of metastatic disease, and use of targeted agents have resulted in an overall decline in mortality, including mortality from distant disease [4, 16]. Several new agents have received FDA approval and are now part of the standard treatment options. Bevacizumab is an anti-angiogenic agent with strong activity in colorectal cancer in combination with chemotherapy [17, 18]. Despite the possibility that Hispanics have less access to this costly agent, we hypothesize that they may derive less benefit from this agent due to host factors which account for bevacizumab’s mechanism of action. Hispanics are poorly represented in the clinical trials with bevacizumab and associated registries; therefore, information with regards to the benefit of this agent in Hispanics is very limited. A retrospective analysis done by our group suggests reduced benefit from bevacizumab in this population [11]. Data in other ethnicities suggest that benefit from bevacizumab is ethnicity dependent [19], which supports the notion that the biological drivers of disease, as well as benefits from targeted drugs are different among Hispanics.

Although many factors impact the changing trends in mortality, Table 1 shows that the closing of the mortality gap coincides with the timeline of FDA approval for newer biologics (including bevacizumab) for treatment of metastatic colorectal cancers [20, 21].

Additionally, it appears that more Hispanics die from metastatic disease, and that mortality by primary site of the disease is different between Hispanics and NHWs (Table 3). Analysis of mortality trends by stage and race reveals that there are outcome disparities not just between Hispanics and NHWs, but also between Hispanic males and females (Table 2). Furthermore, there appears to be a difference in the patterns of mortality from colorectal cancer between Hispanics and NHWs. Compared with NHWs, a higher percentage of Hispanic males die from colorectal cancer.

Although the limitations of the delivery of care in the US play a significant role in generating these results, the significance of patients’ biological factors cannot be overlooked [22, 23, 24]. Colorectal cancer is a heterogeneous disease with a complex biology and disease presentation. Differences in mortality rates by gender and disease site suggest that colorectal cancer in Hispanics may have different drivers, and therefore, may interact differently with the same treatment [25].

In summary, although Hispanics have a lower incidence and mortality of colorectal cancer, several factors have played a role in reducing their mortality advantage. These factors include: lack of access to highly specialized centers, scarcity of representation in clinical trials, different biology of the disease, and perhaps host. It is important to evaluate the reasons for this gap and develop strategies to minimize disparities [26]. Although race and ethnicity are highly correlated with access to care, it is possible that the major factors impacting mortality outcomes are the biological drivers of disease and differential effects of treatment.

Although SEER data provides valuable information on the overall trends in incidence and mortality, lack of availability of detailed treatment information (including type of surgery, chemotherapy and radiation details and duration, and adherence to treatment) limits the ability to draw any definitive conclusions on the interactions between race and treatment effect. Our study is not an exception to this limitation. Given the low representation of Hispanics in clinical trials and diversity within the Hispanic population, development of national registries may be a solution for better understanding the disease and treatment effects among Hispanics with colorectal cancer and to assess the interplay between race/ethnicity and treatment. Currently, with tremendous efforts to personalize treatment to the tumor characteristics, we should not ignore that race and ethnicity may be an important factor in the delivery of personalized care.

Hispanics have an alarming burden of mortality from colorectal cancer which is growing and deserves further exploration. Collaborative efforts to better understand the disease in Hispanics and interventions targeted to this population should be prioritized.

## MATERIALS AND METHODS

To detect disparities and calculate colorectal cancer mortality rates among Hispanics and the NHW population, we used the Incidence-Based Mortality database of the Surveillance, Epidemiology, and End Results (SEER-13) Program. For this purpose, SEER-13 database (1992-2011) was selected. The SEER-13 registries include Atlanta, Connecticut, Detroit, Hawaii, Iowa, New Mexico, San Francisco-Oakland, Seattle-Puget Sound, Utah, Los Angeles, San Jose-Monterey, Rural Georgia and the Alaska Native Tumor Registry, which cover approximately 11.5% of the NHW and 18.8% of Hispanic population. SEER-9 research data (1973-2011) was not used due to the lack of Hispanic ethnicity identification. SEER-18 research database (2000-2011) was not chosen due to the short follow-up time to observe temporal patterns of colorectal cancer mortality rates by racial groups.

Incidence-Based Mortality (IBM) overcomes limitations of other measures of progress against cancer, such as incidence and survival rates, that can be influenced by biases from new screening or imaging techniques. Therefore, IBM was chosen as the end-point of this analysis.

We only chose the first matching cases with microscopically confirmed colorectal adenocarcinoma (primary tumor site: C18.0-C18.9, C19.9, and C20.9, histologic type ICD-03-3 of 8000-8152,8154-8231,8243-8245,8250-8576,8940-8950,8980-8981) who died of colorectal cancer. Deaths reported on autopsy or death certificate only, or cases with age at death younger than 20 years were excluded. Hispanic cases were identified as ‘Spanish-Hispanic-Latino’ from the Hispanic origin code. NHW cases were identified as ‘Non Spanish-Hispanic-Latino’ from the Hispanic origin code and ‘White’ from the race code.

Joint point regression program was carried out to examine the annual trends of stage-specific colorectal cancer mortality rates from 2000 to 2011 among Hispanics and NHWs. SEER historic stage A: localized, B: regional, and C: distant was used for staging. IBM in two periods: 2000 to 2005 and 2006 to 2011 was calculated by race and stage to indicate the temporal change. The relative percent change in IBM between two periods was computed by 100% × (IBM in 2006 to 2011-IBM in 2000 to 2005)/IBM in 2000-2005. Disparity in IBM between the two racial groups in each period was computed by the difference (IBM in NHW-IBM in Hispanics) by stage and overall. The contribution to overall disparity was computed by 100% × Disparity in a stage/Disparity overall [27].

All analyses were conducted using SEER^*^Stat 8.1.5 and Joint Point Regression Program, Version 4.1.0. April 2014.
